# Using positionality and reflexivity to support equity in partnership‐driven research

**DOI:** 10.1111/cobi.14396

**Published:** 2024-11-25

**Authors:** Rachael Cadman, Alanna Syliboy, Michelle Saunders, Shelley Denny, Mary Denniston, Eleanor Barry, Breanna Bishop, Shannon Landovskis, Megan Bailey

**Affiliations:** ^1^ Marine Affairs Program Dalhousie University Halifax Nova Scotia Canada; ^2^ Mi'kmaw Conservation Group The Confederacy of Mainland Mi'kmaq Truro Nova Scotia Canada; ^3^ Nunatsiavut Government Nain Newfoundland and Labrador Canada; ^4^ Unama'ki Institute of Natural Resources Eskasoni Nova Scotia Canada; ^5^ Department of Ocean Sciences Memorial University St. John's Newfoundland and Labrador Canada; ^6^ Department of Oceanography Dalhousie University Halifax Nova Scotia Canada; ^7^ Ocean Tracking Network Dalhousie University Halifax Nova Scotia Canada

**Keywords:** Mi'kmaki, Nunatsiavut, project governance, reflexive methods, research sovereignty, 反思性方法, 项目管理, 研究主权, 米克马克, 努纳齐亚武特地区

## Abstract

Social and economic position and power shape everyone, including scientists and researchers. The way researchers do conservation science and the voices centered in the process are a result of researcher upbringing, experiences, access to resources, and values and are a manifestation of positionality. Positionality is a concept that can help one think about one's position and power in one's work. Creating a successful research partnership requires careful thinking about how equity, diversity, inclusivity, and accessibility are accounted for in the research environment. We drew on our own experiences as early career, mid‐career, and Indigenous researchers to explore researcher positionality and how understanding one's positionality can bring to the fore power dynamics in conservation science and research. We focused on the use of reflexive practice to recognize diverse roles and responsibilities, build strong project governance, and enrich relationships. We considered 2 large research partnerships, Apoqnmatulti'k (Mi'kmaw for *we help each other*) and the SakKijânginnaniattut Nunatsiavut Sivunitsangit (Inuttitut for *Sustainable Nunatsiavut Futures*) project, to examine moments of tension and interrogation of power and the ways in which this interrogation led to stronger relationships and better research. We advise that large transdisciplinary and cross‐cultural research teams use positionality and reflexivity to explicitly make choices about power dynamics in the context of executing partnership‐driven work. This can be accomplished through personal and collective interrogation of the power dynamics at play in project administration, research questions, and interpersonal relationships.

## 利用立场性和反思性支持伙伴关系驱动型研究中的公平性

【摘要】 社会和经济地位与权力影响着每个人, 包括科学家和研究人员。研究人员从事保护科学研究的方式以及在这一过程中发出的声音, 是研究人员的成长经历、经验、资源获取途径和价值观的结果, 也是其立场性的体现。立场性的概念可以帮助人们思考自己在工作中的立场和权力。建立成功的研究合作伙伴关系需要认真思考如何在研究环境中考虑公平性、多样性、包容性和可获得性。本研究以作者们自身作为职业生涯早期、中期和土著研究人员的经历为基础, 探讨了研究人员的立场性问题, 以及了解自己的立场性如何能够体现保护科学和研究中的权力动态。我们的重点是利用反思性实践来认识不同的角色和责任, 建立强有力的项目管理, 并丰富人际关系。我们分析了两个大型研究合作项目, 即Apoqnmatulti'k(米克马克语, 意为我们互相帮助)和SakKijânginnaniattut Nunatsiavut Sivunitsangit(因纽特语, 意为可持续的努纳齐亚武特未来)项目, 以研究关系紧张和权力质询的时刻, 以及这种质询如何促成更牢固的关系和更好的研究。我们建议大型跨学科和跨文化研究团队在开展合作驱动型工作时, 利用立场性和反思性明确做出有关权力动态的选择。这可以通过对项目管理、研究问题和人际关系中的权力动态进行个人和集体质询来实现。**【翻译:胡怡思;审校:聂永刚】**

## INTRODUCTION

As the effects of climate change and biodiversity loss have become evident, scientists are increasingly emphasizing the importance of diverse disciplines and knowledge systems to address social–ecological problems (Bennett et al., 2017; Norström et al., 2020; Syddall et al., 2021; West et al., 2020). Increasingly, this has included research partnerships with Indigenous peoples, who hold essential knowledge and capacity for conservation science (Adams et al., 2014; Dawson et al., 2021; Hoffman et al., 2021; Maclean et al., 2022).

Partnerships between the academy and Indigenous peoples present a unique set of circumstances to the researchers involved (e.g., Gordon (Iñupiaq) et al., 2023; Pedersen et al., 2020). Indigenous peoples are rights holders with sovereignty over research on their traditional territories, which means the historical approach to research on Indigenous communities must be abandoned for research that upholds Indigenous sovereignty and is conducted with Indigenous people as leaders (Bull, 2019; TallBear, 2014). Indigenous peoples are the stewards of their territories and have developed rich knowledge and governance systems over generations that support the health and resilience of the land. We use the term *knowledge systems* to refer to knowledge beyond the facts and methods that often characterize the term. A knowledge system is inclusive of the institutions, practices, and values that inform it (Orlove et al., 2023). To respect diverse knowledge systems and Indigenous research sovereignty, research collaborations must recognize the significant capacities present in Indigenous communities and aim for reciprocal learning that draws on and contributes to both knowledge systems (Alexander et al., 2019).

Working across cultures, languages, and knowledge systems requires researchers to reckon with their own biases and values concerning the nature and purpose of conservation science. Collaborative research that includes diverse voices is inevitably affected by power differentials among the group and affects the motivations, processes, and outcomes of science (Lahsen & Turnhout, 2021; Reo et al., 2017). Colonialism has had a profound impact on the institutions and methods of academia, such that it is embedded in and perpetuated by many researchers’ assumptions, actions, and outputs (Battiste, 2007; Kovach, 2019). All researchers, including ourselves, are implicated in this discursive power imbalance, even when it is subconscious. There is a need for tools that can help Indigenous and non‐Indigenous research teams achieve more equitable and diverse research. Some teams are turning to positionality and reflexive practices, which require researchers to assess personal values and social context to help guide them to more just research processes (Folkes, 2023; Pienkowski et al., 2023).

We work in research partnerships between universities and Indigenous‐led organizations and at the interface between Western science and Indigenous knowledge. We drew on our experiences with positionality and reflected on its possible use as a tool to support successful research partnerships. We considered how we navigated barriers, tensions, and difficult choices in ways that ultimately improved our work but were not without conflict and discomfort. These findings include the need for improved capacity sharing, explicit project governance from the outset, and nurturing peer and mentor relationships between team members.

## BACKGROUND

### Positionality and reflexivity

The word *positionality* comes from anthropology and sociology and applies to ethnographic researchers using reflexive practices to examine their own implicit assumptions (Acevedo et al., 2015). This practice is helpful for pinpointing biases so that researchers can avoid making moral judgements in data interpretation based on their own viewpoints and avoid assumptions about cultures (e.g., gender roles) in research design (Holmes, 2020). However, researchers from marginalized communities point out that the value judgments guiding what research is considered good are dictated by the most powerful segments of society—White, wealthy, male‐dominated institutions, such as universities (Secules et al., 2021). To challenge this, positionality can be used to describe a researcher's social position and power relative to their area of study, directing the researcher to reconcile the disparity between themselves and their research subjects (Brown, 2022). Positionality and reflexivity then become tools for creating more equitable research processes and outcomes.

One of the most frequently employed tools is the positionality statement. Researchers use positionality statements to explain who they are in relation to their work. This includes biographical and personal information, affiliations, and personal motivations. It is an outward‐facing communication tool that helps researchers add transparency to their scientific processes and reporting (Boyce et al., 2022; Hauser et al., 2023). A positionality statement offers the audience a glimpse of the research at a fixed moment in time—it explains who the researchers were and what they believed while conducting the research.

Reflexivity, by contrast, is an active and inward‐facing practice, in which researchers continuously revisit their positionality, power dynamics, and assumptions for the purposes of improving research process and outcomes. Reflexive practice requires researchers to pinpoint their views and values regarding the research process to better assess how their views might shape their research questions, design, and interpretation (Kitagawa, 2023). Reflexive methods may include journal writing, program evaluation, and relationship building activities. Reflexive practice informs how researchers express their positionality (Holmes, 2020). Both positional and reflexive practices can be done as personal, interpersonal, or collective activities (Nicholls, 2009).

Over time, reflexivity and positionality have been adopted outside the social sciences; the methods are now being applied globally (Hauser et al., 2023; Hausermann & Adomako, 2022; Mukherjee, 2017) in diverse areas of research, including computer sciences (Cambo & Gergle, 2022), medicine (Geiser et al., 2022), and conservation science (Artelle et al., 2019; Boyce et al., 2022). Conservation sciences encompass ecological and human systems, so research in this field engages in value‐laden considerations of what is to be conserved, how, and for whom it is conserved (Bennett et al., 2017). Diverse cultures, knowledge systems, and disciplines understand the answers to these questions in unique ways, so research programs may experience conflict when there are multiple values and interests at play.

In North America, the conservation sciences have grown out of colonialist values and norms. For example, pristine and untouched wilderness is valued and used as a rationale to remove Indigenous peoples from their lands and prevent them from managing their resources (Artelle et al., 2019; Bhattacharyya & Larson, 2014). Many have pointed out that equitable research requires a recognition of the values inherent in conservation sciences and planning (Alegado et al., 2021; Lewis, 2012; No'kmaq et al., 2021; Snook et al., 2020). It is argued that by increasing literacy in how values shape research, scientists will be better equipped to anticipate conflict and welcome opportunities for diverse knowledge and value systems to inform conservation sciences (Boyce et al., 2022).

Given this interest in diversifying the values that guide conservation, reflexivity and positionality may be used by researchers seeking to engage with multiple knowledge systems, including those engaged in partnership‐based research. These research teams face a unique set of challenges. In the Western world, colonialism is not only a historical event but also the foundation of contemporary academic research institutions (Kovach, 2009; Latulippe, 2015). Universities have expectations about how research is funded and how data are owned and shared, which often do not align with the expectations of Indigenous communities (Kovach, 2019; McGregor, 2018). University researchers often extract, commodify, and distort Indigenous knowledge through the course of their research in ways that uphold their own values and priorities (Coombes et al., 2011; Latulippe & Klenk, 2020; Reo et al., 2017; Whyte, 2018). Even radical collaborative research partnerships that attempt to address these uneven power relations can end up reinforcing existing colonial structures (Latulippe, 2015) or revert to conventional neoliberal approaches to conservation (Coombes et al., 2012). Given rapidly growing interest in this kind of research partnership, clear guidance is needed to ensure that programs are designed and executed in ways that advance Indigenous sovereignty in research and help individuals navigating these issues.

### Partnership‐driven research

We centered our discussion on what we have termed *partnership‐driven research* to describe where this research sits on the broad spectrum of community‐based research. Where community‐based research can refer to many kinds of research that involve researchers and civic representation, partnership‐driven research is characterized by institutional involvement, where research activities that connect to community are mediated through an Indigenous institution, such as a land claim government, management board, council of Elders, community government or leadership, or corporation. We distinguished this institutional partnership from other forms of community‐based research because these organizations bear a formal or legal responsibility of representation to Indigenous communities and nations. In the context of Indigenous and non‐Indigenous research partnerships, this is one way of conducting work that can uphold Indigenous sovereignty over research.

Working on a partnership‐driven team offers an opportunity to work on research that is led by Indigenous organizations, who can direct projects to be applicable to their context. However, in doing so, researchers must work with multiple knowledge systems and cultures and consider how multiple ways of knowing can inform their work (Carroll et al., 2022; Mercer & Ovitz, 2023). Working across knowledge systems requires careful consideration so that the dominant Western knowledge system does not overpower or subsume Indigenous knowledge systems as secondary because to do so would be to miss important knowledge to guide better research practices and conservation outcomes (Cadman, Dicker, et al., 2023; Reid et al., 2021).

Clear delineation of project leadership, governance, and duties is an essential aspect of partnership‐driven research (Mosurska & Ford, 2020). In each stage of the research process, both institutions are coleaders who are prepared to share their capacities and strengths. Clarity and transparency around research governance is a prerequisite to equitable environmental conservation initiatives (Springer et al., 2021). For example, Reo et al. (2017) found that some collaborative environmental research projects lack respect for Indigenous customary laws and cultural imperatives in the way they carry out their work, falling back into expectations of nature as a provider of resources. Formal agreements on project values and governance can help clarify these types of cultural mismatches (Beveridge et al., 2021; Cadman, Snook, et al., 2023; Castleden et al., 2017).

The creation of a research partnership does not automatically mean that all inequities are solved. Distinct differences in capacity, experience, and institutional support likely still maintain power differentials between partners. Rather, the partnership creates an opportunity to confront inequities as they arise against a backdrop of the existing power dynamics, different sets of cultural expectations, and practical needs. Academic partners must be willing to interrogate and revise their expectations about timelines and deliverables wherever possible because power cannot truly be shared in partnerships where timelines and outputs are determined by academic cultural norms and expectations (Liboiron, 2021; Reed & Rudman, 2022). Beyond the boundaries of the project itself, the broader institutions that govern research will ultimately still affect how much power and responsibilities can be shared among parties.

Indigenous partners still face barriers that academic institutions do not, for example, not being allowed to hold and manage funding for the project, narrow conceptions of what constitutes expertise and seniority, unequal division of labor, or discrepancies in how ethical requirements are evaluated. We explored some of our own experiences with these institutional power imbalances, focusing primarily on the formal and informal structures within our research partnerships and our interpersonal journeys therein.

## CASE STUDIES

### SakKijânginnaniattut Nunatsiavut Sivunitsangit

Nunatsiavut is one of 4 regions of Inuit Nunangat, the Inuit homelands in the Arctic and Subarctic of Canada. The region was formed under a Comprehensive Land Claim Agreement in 2005, which recognizes Labrador Inuit title of over 72,520 km^2^ of land and an additional 48,690 km^2^ of tidal waters. The Labrador Inuit Land Claims Agreement led to the creation of the Nunatsiavut Government (NG), an Inuit self‐government with responsibilities over health, natural resources, education, culture, and economic development (White & Alcantara, 2020).

There is a long history of extractive research practices in northern Canada, in which researchers have come into communities or onto their lands, taken information, samples, and insights, and left without coordinating with community members, sharing their results, or using their findings to create change in people's lives (Laidler, 2006; McGrath, 2018; Pedersen et al., 2020). Many Inuit researchers have commented on the frustration and harm caused by these modes of research (Pfeifer, 2018; Tester & Irniq, 2008). Because of this history, Inuit Tapiriit Kanatami, a nonprofit organization representing Inuit across Canada, developed a strategy to advance Inuit sovereignty in research, the National Inuit Strategy of Research (NISR). The NISR outlines 5 priorities for research in Inuit regions: advance Inuit governance in research; improve researcher's ethical conduct; redirect research funding to Inuit priorities; develop Inuit sovereignty over data and information; and build capacity in Inuit Nunangat (Inuit Tapiriit Kanatami, 2018). This guidance provides a framework for any researcher hoping to work in Inuit Nunangat.

The NISR emphasizes the importance of Inuit acting as leaders in research. In that spirit, principal investigators in the NG and from Dalhousie and Memorial Universities are coleaders of the SakKijânginnaniattut Nunatsiavut Sivunitsangit (Sustainable Nunatsiavut Futures) (SNF) project. The objectives for the project were codesigned for the original funding application. The project began in 2019 and involves over 50 individuals, including academic researchers, government (federal and Inuit) researchers, nongovernmental organizations, and community members from Nunatsiavut. Team members study the physical, chemical, biological, and ecosystem functions of coastal and marine waters. The project brings together natural and social scientists and Inuit Knowledge holders to identify changes in the marine environment and collaboratively plan for ways of adapting to or mitigating these changes. Through this research, the SNF project hopes to uphold the NISR vision and support Inuit research sovereignty.

### Apoqnmatulti'k

Mi'kma'ki is the traditional unceded territory of the Mi'kmaq, stretching across Atlantic Canada and Quebec. The Mi'kmaq are one of the signatories to the Peace and Friendship treaties, which were created during the 18th century and recognize Mi'kmaq as a sovereign people (Denny & Fanning, 2016). At the time of European contact, Mi'kma'ki was divided into 7 districts: Unama'kik, Epekwitk aq Piktuk, Eskikewa'kik, Sipekni'katik, Kespukwik, Siknikt, and Kespek. Each was led by a hereditary district chief. Mi'kmaq were also members of a larger political organization known as the Wabanaki Confederacy, which also included Abenaki, Passamaquoddy, Penobscot, and Wolastoqey. From the 16th century onward, colonizers created numerous borders, laws, and institutions that drastically changed governance in Mi'kma'ki, such that today there are communities in each province organized as “bands” under the *Indian Act* (13 in Nova Scotia, 9 in New Brunswick, 2 on Prince Edward Island, 3 in Newfoundland and Labrador, 3 in Quebec, and 1 in Maine). These bands are led by elected chiefs and councils.

Several Mi'kmaq organizations have become leaders in conservation sciences in Nova Scotia. Much of this work is driven by the Mi'kmaw epistemological concept of M'sɨt No'kmaq (i.e., all my relations [kin‐relationship between humans and the earth, where humans are an equal part of the lands, waters, plants, and animals that sustain them]) (Denny & Fanning, 2016; No'kmaq et al., 2021) and by the concept of Etuaptmumk (2‐eyed seeing). Etuaptmumk was introduced by Elder Albert Marshall in 2003 to describe a mode of inquiry in which both Indigenous and Western knowledges are recognized and respected and woven together for research that is based on partnership and mutual benefit (No'kmaq et al., 2021). Mi'kmaw research institutions, such as the Confederacy of Mainland Mi'kmaq (CMM) and Mi'kmaw Conservation Group (MCG) and Unama'ki Institute of Natural Resources (UINR)—2 Aboriginal Aquatic Resources and Oceans Management groups (AAROMs)—ground their research in these principles for the stewardship of the lands and the advancement of Mi'kmaq sovereignty.

Apoqnmatulti'k (Mi'kmaq for *we help each other*) is a partnership between the CMM and MCG, UINR, Marine Institute of Natural and Academic Science, Fisheries and Oceans Canada, Ocean Tracking Network, Acadia University, and Dalhousie University. The research objectives for this project were codesigned by project partners who recognized the critical state of aquatic ecosystems and shared a desire to learn from each other and work together to better understand and steward them. The project studies culturally valued aquatic species, including katew (American eel [*Anguilla rostrata*]), jakej (American lobster [*Homarus americanus*]), ji'kaw (striped bass [*Morone saxatilis*]), and punamu (Atlantic tomcod [*Microgadus tomcod*]) in the Pekwitapa'qek (Bay of Fundy) and Pitu'pa'q (Bras d'Or Lake). Their research focuses on acoustic telemetry studies that are cocreated by academic and Indigenous scientists and knowledge holders. Research activities are conducted in accordance with Mi'kmaw ecological values and for the benefit of functioning coastal ecosystems and the people who rely on them. The project began in 2018 and includes over 20 researchers, administrators, and community leaders.

## AUTHOR POSITIONALITY

The following is a brief explanation of who we are as individuals in relation to the 2 case studies. Rachael Cadman is a settler originally from Guelph Ontario, the traditional territories of the Anishinaabeg and Haudenosaunee, on the land of the Mississaugas of the Credit First Nation. She works to advance just outcomes in marine spatial planning and is affiliated with the SNF research program. Alanna Syliboy is a member of the Sipekne'katik First Nation and is the community liaison officer for both the Department of Environmental and Natural Resources at the CMM and the Apoqnmatulti'k program. Michelle Saunders is an Inuk woman, originally from Happy Valley‐Goose Bay, Newfoundland. She is the research manager at the NG in Nain, Newfoundland, and was motivated to work in conservation after experiencing a lack of Indigenous representation in traditional conservation spheres. She is a partner on the SNF program. Shelley Denny is a Mi'kmaq woman from Potlotek First Nation and the director of Aquatic Research and Stewardship at the UINR. She holds a PhD from Dalhousie University and is a partner on the Apoqnmatulti'k research program. Mary Denniston is an Inuk Woman from Nain, Newfoundland and Labrador. She completed her BA at Grenfell College and is a partner on the SNF research program. She is motivated to work in conservation because she is able to practice her people's connection and keep her people's values and traditional ways of living alive by respecting and guarding her people's land. Eleanor Barry is a settler originally from Lothersdale and Alderton in the United Kingdom. She is a first‐generation college student, attending the Memorial University of Newfoundland and Labrador. Her doctoral work is associated with the SNF project and focuses on plankton dynamics and food web interactions linked with sea ice. Breanna Bishop is a settler of European descent, originally from Hornby Island, British Columbia. She is a PhD candidate at Dalhousie University affiliated with the SNF research program. Her work is underpinned by a desire to work in partnership with Indigenous peoples on whose lands research takes place. She currently studies how Inuit Knowledge can reshape Western scientific approaches to understanding the marine environment in Nunatsiavut. Shannon Landovskis is a non‐Indigenous woman of European descent, originally from Palgrave, Ontario. She completed her Master of Science degree as a member of the Apoqnmatulti'k team and is now a field operations and data acquisition coordinator for the Ocean Tracking Network. She works in this field to contribute to work that gives back to the earth and the species that share it with humanity. Megan Bailey is a non‐Indigenous woman originally from London, Ontario, which is situated on the traditional territories of the Anishinaabeg, Haudenosaunee, Lūnaapéewak, and Attawandaron, and is an associate professor at Dalhousie University. Her work focuses on equity in fisheries governance, with a particular emphasis on allocations and benefits distribution. She is a member of the Apoqnmatulti'k and SNF research programs, primarily as a supervisor of student and postdoctoral fellow work and as a member of project steering committees.

## REFLECTIONS ON SITES OF INEQUITY AND POSITIONALITY

In these reflections, our distinct positionalities place us in different relationships with each other, with the communities in which we work, and with the land. By being transparent with our positionalities, we located areas of tension and difference and used reflexive practices to address them. This section draws on the many conversations and meetings between members of these 2 groups through the course of working together—since 2018 for members of Apoqnmatulti'k, and since 2019 for members of SNF. We expanded on personal narratives throughout. The themes we addressed illustrate how these research partnerships were informed by our individual and collective positionalities (Maclean et al., 2022).

Reflective sessions were led by Rachael Cadman with each project team to develop the main concepts and themes shared here. The groups were prompted using discussion questions on the nature of their partnerships, the reflexive practices they have engaged in, and the enablers and barriers they face in working together. Themes were developed out of these conversations, which included summarized ideas from the discussions and some direct quotes from speakers. These were presented to all authors, who added further comments, clarifications, and relevant anecdotes.

## PARTNERS UNDERSTANDING OF ACCOUNTABILITY

Both teams found that positionality was important to foster a sense of accountability within their research teams. Shelley points out that Apoqnmatulti'k took this literally in their name—partners agreed to help each other, and that drives the work they do. Accountability has many directions: partners are responsible to each other, to the community they work with, to project leadership, to funders, and to students and mentees. For Indigenous partners, the responsibility to their own communities has significant consequences. Michelle explains “I am inherently more responsible to the community than people from the south—there are consequences for me if something bad happens, in my personal life and in my work.” It is important for non‐Indigenous team members to understand what is at stake for Indigenous team members. Because non‐Indigenous researchers, who are not part of the communities they are working with, do not experience the same responsibility, teams must find explicit ways of fostering accountability by recontextualizing the relationship between researchers and communities.

All researchers on Apoqnmatulti'k worked and lived in Mi'kma'ki, which meant they were able to immerse themselves in the cultural context of the work. Researchers participated in events and ceremonies that helped them build interpersonal bonds and a deeper sense of accountability to the land and communities. Led by Alanna, Apoqnmatulti'k starts monthly team meetings with a reconciliation in action reflection or activity. Calendar invites are shared for upcoming cultural activities (e.g., Fall 2023 salmon celebration), or thanks is given for participating in something together (e.g., a pow wow for a Mi'kmaw community with ties to the project). These activities help non‐Indigenous researchers build relationships in the community and across the partnership team.

In SNF, most academic researchers live outside Nunatsiavut, so frequent and prolonged stays in community have helped early career researchers develop relationships outside the sphere of research, leading to a deeper understanding of the ways they were guests in the region and of the implications of such positionality. Eleanor arrived in Nunatsiavut for the first time in 2023. She said, “My extended stay allowed me to build meaningful relationships and understand the stakes of the work, and who I am accountable to.” Mary shared the following: “If you want to do good research here, you have to come and meet the people who will be doing a lot of the groundwork for you, but also to understand that this is home to many people.” Non‐Indigenous partners have a responsibility to ensure the Indigenous people and culture are respected and engaged.

Indigenous research partners are often taking on more risk in joining these research teams than their non‐Indigenous colleagues. They risk having their knowledge systems and their expertise dismissed or undervalued, potential harm to their communities, and damage to their reputations and personal relationships. They face these risks repeatedly with each research activity or new project member. Using reflexive methods as a way of confronting this power dynamic can help team members understand what is at stake in their work. To do this, non‐Indigenous researchers must spend time in community and develop personal relationships to better understand the stakes of their actions and to help reorient them to work in service of the community.

## CELEBRATING DIVERSE EXPERTISE

Every research project requires a diversity of roles, expertise, and tasks: grant writing, project administration, teaching, data collection, data analysis, writing, community liaisons, public communications, and mentorship. Transparent conversations about positionality can help team members reflect on their expertise and their roles in the project. However, Shelley observed that often Indigenous partners are seen exclusively as advisors—a resource to be drawn on: “We are never seen as experts until we are needed. A lot of time we feel used, people get money and credit and then they go away.” Research partnerships should recognize and make room for the many roles of Indigenous partners. This is particularly true in the context of research. Many Indigenous partners are researchers and scientists, even though they often get labeled as *partner*, rather than *scientist*. Through our discussions, we identified 3 important roles that Indigenous team members have played that have been essential for successful partnerships: mentorship, translation, and safeguarding. These roles had a direct effect on the power dynamics within the teams.

Throughout the research process, both the Apoqnmatulti'k and the SNF teams have made use of informal mentorship between senior Indigenous team members and junior researchers. Shelley has been an important mentor for Alanna on the Apoqnmatulti'k project, providing guidance on what it means to navigate the academic university as a Mi'kmaw woman. Indigenous mentors can lend strength to early career Indigenous researchers and equip them with the tools they need to succeed. Mary and Rachael developed a mentor–mentee relationship, with Mary modeling what it means to be accountable to the community. She taught Rachael that research can be a tool for cultural rejuvenation and celebration. Mentorship was important in both cases in helping these researchers better understand their own relationship, skills, and responsibilities to the work.

Indigenous team members played an important role in translations between academic and Indigenous cultures. Shelley “is the prime example of Etuaptmumk—a Mi'kmaw woman trained in science” (Alanna). She brings so much of what she has learned from her elders and knowledge holders in her community and beyond to inform the science of Apoqnmatulti'k.

As well as a bridge, Indigenous team members form an important boundary between their community and researchers and can limit researchers’ access to their communities. Michelle notes that Nunatsiavut Research Centre staff evaluate research ideas to ensure that proposals are in line with Nunatsiavut priorities and that potential conflicts or tensions are avoided. This work is done informally, through meetings or phone calls, and through the Nunatsiavut Government Research Advisory Committee, which reviews research proposals and ethics applications. They bring this expertise to every planning session on SNF research projects and can advise on essential considerations, such as work already being done in the region, gaps and priorities for research, ethical problems, and cultural misunderstandings emerging in ongoing research.

These roles are essential for developing research programs that have relevant questions, ethical processes, and beneficial outcomes, and yet they are not always valued in terms of the knowledge, skill, and experience they require. Certain types of labor remain invisible without open, transparent reflections. Positionality can help teams develop better awareness of the types of labor involved in partnership‐driven research.

## BUILDING STRENGTH THROUGH STUDENT SPACES

In both projects, we found that student groups offered open‐minded and supportive places where reflexivity and positionality could be explored together. The SNF has a student group called IlinniaKatigennik (*learning together*), where students, post docs, and Inuit research coordinators meet monthly to share updates on their research and their personal lives. From our perspective (Breanna, Eleanor, and Rachael), the group has been a positive space for students to find context on the project and the region and find overlaps between their research programs across disciplines. Breanna comments that “the supportive nature of the group allows us to learn from one another in a space that encourages us to express thoughts, ask questions, and challenge ideas in a way that effectively communicates across different ways of knowing.” Throughout her time with Apoqnmatulti'k, Shannon found that willingness to share in discomfort, both when pointing out the actions of others and when being called out, had a large effect on her work. Sitting in discomfort and learning from it is an act of generosity and shows a level of commitment to the project and to the people in it and contributed significantly to building an environment where partners could feel safe and valued enough to continue to contribute.

Student spaces were also effective in helping Indigenous and non‐Indigenous early career researchers develop skills around communicating with senior leadership. The student subcommittee of Apoqnmatulti'k was a supportive environment for Alanna, who said: “I could ask a question and not feel stupid about it. Sometimes I felt less equivalent in the larger group. The early career folks felt more open‐hearted and open‐minded. It builds confidence when you have that backing.” Alanna was able to voice her opinions to the student group, and with their encouragement she felt confident in bringing her feelings to the larger group. Megan recalls that “Alanna called me out when our team was arguing about hole‐punching the tails of Sipekne'katik‐caught lobsters. I was upset and embarrassed because I thought I was doing and saying the right things. So, I had to try to understand how my positionality was the very reason there were no right things for me to say, it simply was not for me to talk about. After our meeting ended, I called Alanna on the phone to apologize and to thank her.” This feeling of support is important for Indigenous partners and students to understand their contributions as valuable, which helps keep the project accountable and can lead to more truly collaborative outcomes. Early career research groups can lend great strength to a partnership‐driven project because they offer new researchers a place to develop skills in exploring reflexivity and positionality among peers.

## STRONG PROJECT GOVERNANCE

In traditional academic programs, individual actions are prioritized, in part because they help build curriculum vitae and push scholarship forward. However, in partnership‐driven work, accountability is a collective good. A project can and should be seen as an institution in and of itself. It sets the rules and norms, what is rewarded, and what is not rewarded. In SNF, despite an intention to do science differently, project structures to support this aim were not put in place early in the project. Megan notes, “Bite‐sized chunks were tackled, like a values elicitation process led by Max Liboiron, coauthorship guidelines led by Mike Petriello, and the formation of IlinniaKatigennik. But those pieces did not feed into any larger governance structure where power could be re‐evaluated. Seemingly, hierarchical academic leadership as the default option crept back in, leading to mistakes being made and relationships being challenged.” Noting this limitation, SNF project leaders embarked on a reform initiative to improve knowledge mobilization, project legacy, and capacity sharing, recognizing that it is never too late to improve governance.

Students need guidance to navigate these difficult spaces. Eleanor highlights the “importance of check‐in points to talk about our positionality and our motivations, to ensure that we're still doing what we set out to do. Those conversations would help me understand my work differently and would bring more transparency to the project over all.” Similarly, Michelle reports feeling pressure from students and senior academics: “without governance structures or research review processes in place, Indigenous partners often feel pressure to speak on behalf of their community and region. Although I am one person living in Nunatsiavut, I cannot speak on behalf of all of Nunatsiavut.” In Apoqnmatulti'k, the student experience was similar. Because of COVID‐19, critical relationship building and knowledge sharing opportunities were not held, which greatly affected the students’ ability to engage with the community they were working with. Despite this, the overarching goals outlined for the students remained unchanged, and students felt pressure to navigate this without organized support from senior project partners. Ensuring governance structures are in place to evaluate the process early and often relieves the burden of capacity and representation from a few individuals working within these partnerships to a more diverse and more representative group from communities. Importantly, this includes self‐ and group evaluations from project teams to reflect on personal and collective positionalities.

## PERSONAL AND INTERPERSONAL REFLEXIVITY

Reflexivity can be used to examine personal actions and motivations and to facilitate relationship building. Examining personal biases, maintaining honest conversation, and admitting mistakes require a lot of vulnerability, and this is not a skill that comes naturally to everyone. Research has ethical and emotional risks for researchers as well as participants, as is the case when researchers are asked to publicly share and confront those vulnerable places (Shaw et al., 2020). This is about more than allowing oneself to look bad—it is about confronting the systems of power and marginalization that are present in our actions (Anthony‐Stevens & Matsaw Jr, 2020). Tuck and Yang (2012) speak about the “settler move to innocence,” an “attempt to deflect a settler identity, while continuing to enjoy settler privilege and occupying stolen land” (p. 11). Settler researchers must be prepared to grapple with the ways they uphold and perpetuate colonial power and actually give up some of their power (Held, 2020). The feelings of risk and discomfort experienced by many when they start to think through their positionality lie in this realization. Individuals should engage in this kind of personal, introspective, and emotional work before joining a project, to decide whether they belong, and what roles and responsibilities they hold if they do participate.

Our reflections draw attention to how the default position for Indigenous colleagues is in an advisory role, minimizing the important work they do as teachers and mentors, researchers, community representatives, bridges to community, and stewards. By engaging in reflexive practices, we came to understand the importance of regularly returning to our interpersonal positionality—to recognize the diversity of roles that make these research programs possible and effective. This includes building in time and funding for these activities, for example, dedicated support for Indigenous mentor–mentee relationships, independent early career researcher spaces, professional development training, and adequate remuneration of labor (Enright & Facer, 2017; Sawyer et al., 2024).

Mutual learning initiatives can encourage more open, reflexive work in a research partnership. We found that early career research spaces can help engender feelings of trust, support, and growth. Although academia frequently defaults to a hierarchical structure, student groups remain open to peer learning, which was a more comfortable setting for mutual learning and growth. This reinforces findings elsewhere in the literature (e.g., Tondu et al., 2014; Woodward & McTaggart, 2016), where mutual support and understanding are key to early career success in community‐based work.

However, partnership‐driven projects should not count on student groups and Indigenous research partners to be the sole site of all these responsibilities. Professors and others at the top of the academic hierarchy should be prepared to engage with positionality to learn how to use their power to support more open, mutually accountable relationships. For example, professors embarking on partnerships should develop guidelines for themselves on what it means to be a good mentor to prepare their students for proper research conduct with Indigenous partners. These guidelines could be part of a longer exercise in reflexivity and self‐evaluation and be revisited collectively to ensure that professors are continuously relinquishing power.

Moreover, in leading reflexive discussions through trust, accountability, and generosity, we came to understand our relationships as an outcome of the research, not a method to improve our results. Genuine friendships have formed because of our efforts to work together, and we believe this should be a motivating factor to creating partnerships.

## COLLECTIVE REFLEXIVITY

Reflexivity and positionality can also be used as a tool to improve project structure and governance if it is employed early and throughout the life cycle of the project by leaders. This means looking beyond the sphere of individual responsibility. Pressure from funders and from university expectations around timelines and deliverables can force even the best‐intentioned researchers back into prioritizing certain activities and outputs (Held, 2020; Mercer & Ovitz, 2023). Establishing protocols at the outset of a project can help alleviate some tension when difficult questions arise. Culturally relevant research frameworks have been found to empower Indigenous governance and lead to more ethical research (Duke et al., 2021). This requires that project leaders build reflexivity into each stage of the research process so that team members understand one another's roles and responsibilities and highlight continuing power differentials that exist within the group so that the group can address them. Table 1 is a compilation of considerations to make at each stage of the research process to help address some of the tensions and questions that have arisen during our own work in research partnerships. Therein, we also provide guiding questions to help research teams reflect on how to lead functional and equitable collaborations.

**TABLE 1:** Considerations that should be made at each stage in the research process to support equitable research partnerships.

Stage	Consideration	Question
Establish partnership	Research team capacities	What capacities already exist in each team? What additional capacities are needed to work together? Are nonuniversity partners able to hold funding? How are roles and responsibilities divided among partners?
	Research team protocols	Who has authority? What culturally appropriate methods can be used to make decisions (Wilson‐Hokowhitu, 2019)? How will the group address conflict when it arises? What are the expectations for project process and outcomes (e.g., timelines, outputs, ownership, theory of change)? How will the group bring new team members onboard (Chuenpagdee & Jentoft, 2007)?
	Guiding principles	What values does the group share that can guide the work? How will individuals be accountable to those values?
Identify questions	Setting the research agenda	How are research questions and objectives developed (Datta, 2018)? How are research processes and methods designed?
	Applicability of the research to local context	Is the research useful in real time? Is it in line with what the community or partner organizations have identified as a priority (Harmsworth et al., 2016)?
Seek support	Management and distribution of funding	Has funding been built‐in for prioritizing community needs, for research time in community, and for relationship building activities?
	Longevity of the research	Is there sufficient time built into the project for relationship building (Tondu et al., 2014)? Is there funding in place to support long‐term engagement? Are there team members with the stability and permanence to maintain the project over a long time (Pedersen et al., 2020)?
Conduct research	Data collection	Is training available to support capacity sharing among team members (Mercer & Ovitz, 2023)? Are cultural, spiritual, and linguistic considerations accounted for? Is there adequate recognition of labor and expertise concerning logistics, site selection, participant identification, safety concerns, cultural mediation, or translation?
	Storage and management of data	How will data be stored and shared over the long term (Kukutai & Taylor, 2016)? Does the capacity exist to maintain storage? Are there data‐sharing agreements in place? Who will be responsible for making sure agreements are upheld?
Verify results	Accounting for diverse voices and knowledge systems	What happens when there is conflicting information between knowledge systems? Does everyone need to agree on the interpretation of data?
Report findings	Communicating results	What types of outputs will be used to share information? Who will results be shared with? How can communication style and outputs be tailored to meet the needs of the audience? How will authorship inclusion and order be determined (Liboiron et al., 2017)?

## LIMITS OF REFLEXIVITY

Despite the important personal and structural gains that can be done through positionality and reflexivity, these tools are not a panacea to address problems in Indigenous and Western research partnerships. Systemic issues concerning the values, norms, and expectations that govern academia continue to complicate the ability to work together equitably. For example, issues with how research funds can be administered and who is considered a qualified expert to hold research funding continue to complicate the capacity to truly share power. Such issues can put Indigenous cultural and intellectual property at risk, and no amount of self‐reflection will protect it (Artelle et al., 2019). In addition, the extent to which an Indigenous and non‐Indigenous research partnership can encounter the power disparities that characterize these relationships is deeply dependent on context, including the political, economic, social, cultural, linguistic, and spiritual character of the research team. Different geographies, disciplines, and people will encounter this practice in unique ways.

Coombes et al. (2012) comment on the capacity‐building narrative of academic research that always seems to mysteriously lengthen when working in Indigenous territory, such that it is never ending (i.e., communities are forever in need of more capacity; they can never be left to govern themselves). This is a false paradigm, and it devalues the significant capacities and expertise held within Indigenous communities. Conservation science cannot move forward in true partnerships until researchers move from capacity building to capacity sharing (Mercer & Ovitz, 2023) and safeguard Indigenous sovereignty over their research (Carroll et al., 2022). In the long term, truly equitable research partnerships will not only require personal, interpersonal, and collective reflexivity, but also an overhauling of the research culture and institutions such that indigeneity is not displaced in favor of business‐as‐usual colonial research (Smith et al., 2021).

## CONCLUSION

Partnership‐driven work is not easy. In bringing together cultures, knowledge systems, and expertise, one must make explicit variations in approaches, values, and norms. We drew on 2 case studies to outline how articulating our own positionalities and understanding those of others, combined with a reflexive practice, have helped us learn and grow together. In doing so, Indigenous partners and researchers have exercised sovereignty over research processes and practices, such that the conservation science done in these case studies has been done with, for, and by Indigenous peoples. Indigenous research sovereignty requires a shift in power in the way academic partnerships are often carried out, and clear and consistent articulation of the group's relative positionalities is essential for achieving strong shared outcomes. However, personal and interpersonal reflexivity is only one step toward restructuring research institutions to achieve justice for Indigenous peoples in research.

We have found that positionality has been important in confronting some of the power imbalances inherent in partnership‐driven research. We have used reflexive practice to articulate the roles and responsibilities that are frequently unseen and underappreciated, particularly those Indigenous research partners play in mentorship, translation, and safeguarding. We found reflexivity to be useful for facing our personal and collective accountability to one another, to research outcomes, and to the land on which we work. This work is enabled through strong governance from the outset and engaging in self‐ and group reflexive practices throughout the life cycle of the project to ensure that the values and objectives of the partnership are upheld.

We write as a group of individuals, each with our own experience of these projects, which should not be taken for consensus across the partnerships. Others will have experienced the work differently. Scientific publications generally require one unified voice, with all authors in perfect agreement, but maybe having diverse voices means that sometimes we disagree, even when looking at the same data, because our knowledge systems, backgrounds, and experiences (i.e., our positionalities) are different. Maybe homogenizing voices in scientific communication does a disservice to the spirit of diversity in science. We have presented our personal experiences with partnership‐driven research to provide insight into the complexities of working across culturally, socially, and physically distinct spaces.
